# Prevalence and determinants of preconception folic acid use: an Italian multicenter survey

**DOI:** 10.1186/s13052-016-0278-z

**Published:** 2016-07-13

**Authors:** Roy M. Nilsen, Emanuele Leoncini, Paolo Gastaldi, Valentina Allegri, Rocco Agostino, Francesca Faravelli, Federica Ferrazzoli, Enrico Finale, Paolo Ghirri, Gioacchino Scarano, Pierpaolo Mastroiacovo

**Affiliations:** Department of Global Public Health and Primary Care, University of Bergen, Kalfarveien 31, 5018 Bergen, Norway; Alessandra Lisi International Centre on Birth Defects and Prematurity, Rome, Italy; Unit (UOC) Obstetrics and Gynecology, Santo Spirito in Saxia Hospital, Roma, Italy; Unit (U.O.) of Pediatrics, Vaio Hospital, Fidenza, Parma, Italy; Department of Mother and Child Health, S. Giovanni Calibita-Fatebenefratelli Hospital, Rome, Italy; Medical Genetics Unit, Galliera Hospital, Genova, Italy; Unit of Neonatology, Santo Spirito in Saxia Hospital, Roma, Italy; Unit of Obstetrics and Gynecology, Castelli Hospital, Verbania, Italy; Unit of Neonatology and Neonatal Intensive Care, Azienda Ospedaliero-Univesitaria Pisana, Pisa, Italy; Department of Medical Genetics, Gaetano Rummo Hospital, Benevento, Italy

**Keywords:** Folic acid, Italy, Neural tube defects, Predictors, Pregnancy, Supplementation

## Abstract

**Background:**

Women in many countries are advised to use folic acid supplements before and early during pregnancy to reduce the risk of neural tube defects in their infants. This study aimed to update the prevalence and to identify possible determinants of preconception folic acid supplement use in Italian women.

**Methods:**

The study was based on cross-sectional data from seven maternity clinics located in six Italian regions from January to June, 2012. Data on maternal characteristics and supplement use were collected for 2,189 women using a self-administered questionnaire.

**Results:**

Preconception folic acid use was reported by 23.5 % (*n* = 515) of the participants. Of these, 479 (93 %) women had taken folic acid supplements on a daily basis as recommended by the health authorities. Women who both had intended their pregnancy and had requested a preconception health visit to a doctor/gynecologist were substantially more likely than the reference group to initiate folic acid supplementation before their pregnancy (48.6 versus 4.8 %). Preconception folic acid use was also associated with higher maternal age, higher education, marriage/cohabitation, lower parity, infertility treatments, and chronic disease.

**Conclusions:**

Data from seven maternity clinics located in six Italian regions indicate that preconception folic acid supplement use in many Italian women is low. Women who do not plan their pregnancy or do not request a preconception health visit to their doctor have among the lowest prevalence of preconception folic acid use. Improving folate status in these and other supplemental non-users may have important disease preventive effects.

## Background

Health authorities in many countries around the World recommend women of reproductive age to use folic acid supplements before and early in pregnancy to reduce the risk of having a baby with a neural tube defect [1–3]. The recommendations usually indicate a dose of 0.4 mg folic acid daily from at least one month before pregnancy until the third month of gestation. In addition to such recommendations, a number of countries have also introduced mandatory food fortification of grains with folic acid to increase intake of this B-vitamin in fertile women. In these countries, a risk reduction of neural tube defect has been reported [4–7].

Mandatory food fortification with folic acid has not yet been introduced in any European country [3, 8]. Enhanced sources of folate are only available from folic acid supplements, voluntary fortification, and intake of foods rich in folates, such as vegetables and fruits. Although the prevalence of preconception folic acid supplementation in Europe has increased after many years of delivered recommendations, a large portion of women still does not follow the recommendations and many start supplementation to late with respect to neural tube defect prevention [9]. Additionally, the total prevalence of neural tube defects in Europe has not declined markedly over the years [3, 10, 11]. This has led to a debate regarding the efficiency of folic acid recommendations in Europe and other countries [12, 13].

In order to identify groups of women who do not follow the recommendations, many researchers have explored the possible determinants of use or non-use of folic acid supplements. Typically, pregnancy intention, preconception health visit to a doctor, and higher educational level are strong correlates for taking folic acid supplements [14–18], while higher parity, shorter and longer interpregnancy intervals, maternal smoking before pregnancy, and lower maternal age are often associated with less use [15–19]. Such information is valuable and can be used to design intervention programs to improve the preconception folic acid supplement use overall and in subgroups of women related to low use.

In Italy, the official recommendations from April 2004 state that all women planning a pregnancy and those who do not actively exclude the possibility of becoming pregnant should take a daily folic acid supplement of at least 0.4 mg from one month before conception and continue throughout the first trimester [20, 21]. With a prescription from a doctor, this dose or higher is free of charge in Italy since 2005 [22]. Since the folic acid recommendations were issued in 2004, information on the determinants of supplement use in Italian women is limited [14, 23, 24]. This study, therefore, aimed to update the Italian prevalence and to identify possible determinants of preconception folic acid supplement use in Italian women using data from a recent Italian multicenter survey on preconception health.

## Methods

### Study population

The current study drew on resources of a multicenter study initially established to examine the prevalence and the determinants of a variety of preconception risk factors (including preconception folic acid supplement use) in Italian women [25]. It was conducted between January and June, 2012, in seven maternity clinics located in six different regions: Castelli Hospital (Verbania, Piemonte), Vaio Hospital (Fidenza, Emilia-Romagna), Galliera Hospital (Genova, Liguria), Santa Chiara Hospital (Pisa, Toscana), S. Giovanni Calibita Fatebenefratelli Hospital, and Santo Spirito Hospital (Roma, Lazio), Rummo Hospital (Benevento, Campania). The study was approved by the ethical committee of the S. Giovanni Calibita Fatebenefratelli Hospital (parere 55/2011).

The data collection of the present study has been described elsewhere [25]. All women eligible for the study were invited to fill in a self-administered questionnaire comprising 35 initial questions on various topics, including vitamin supplement use, pregnancy-related risk factors, and maternal background characteristics. Personal information such as names, address, or birth date was not recorded. In Verbania (*n* = 320), women were recruited during pregnancy (usually at their first prenatal visits), while in the other six maternity clinics (*n* = 1,892) mothers were recruited during hospitalization after giving birth [25]. Mothers of newborns with any congenital malformation or low birth weight (<2,500 g), with children who were delivered preterm (<37 weeks gestation), or mothers with children admitted to the neonatal intensive care unit were initially not invited to participate to avoid subjecting them to unnecessary stress. Also, women not speaking Italian were excluded from the study.

The distribution of the 2,212 participants at the seven participating maternity clinics was as follows: Verbania (14.5 %), Genova (17.8 %), Fidenza (9.9 %), Pisa (9.1 %), Roma Fatebenefratelli (23.1 %), Roma Santo Spirito (10.8 %), and Benevento (14.7 %) [25]. Of the 2,212 participating women, we excluded 23 women who did not have information of folic acid supplement use (*n* = 5) or did not provide information on the time period of use (*n* = 18), leaving back 2,189 women for analyzes.

### Folic acid supplement use

The women were asked to report data on supplemental use of folic acid and other vitamins by specifying the brand name, the frequency of use, as well as the time period of use. In this study, we defined folic acid supplement use as any use of supplements containing folic acid for at least once a week during the following predefined time periods: (a) at least one month before the last menstrual period to the end of the first trimester, (b) at least six months before the last menstrual period to the end of the first trimester, (c) soon after pregnancy confirmation to the end of the first trimester, (d) after the first trimester of pregnancy, or (e) no use at all. The main outcome variable under study was *preconception* folic acid supplement use, i.e., any use of folic acid supplements that had started before the last menstrual period (category (a) and (b) above). Note that our definition of preconception folic acid use differs somewhat from the official recommendations by allowing for a more moderate use as well, i.e., less than seven days a week.

### Potential determinants

Based on the questionnaire, we abstracted data on the following relevant determinants of folic acid supplement use: maternal age at study recruitment (Verbania) or delivery (other regions), parity, maternal education, marital status, national citizenship, working activity, pregnancy intention, preconception health visit, infertility treatment, maternal smoking, prepregnancy body mass index (kg/m^2^), and chronic diseases. The pregnancy was classified as intended, mistimed or unintended on the basis of a score of the intensity of pregnancy planning effort developed by Morin et al. [26] The following categories were used: intended (score 9–12), mistimed (score 4–8) and unintended (score: 0–3). The preconception health visit was defined as any visit to a doctor/gynecologist within the last year before the onset of pregnancy to ask for any advice on the possible near future pregnancy, such as medical examinations to perform, medications and vitamins to take. Body mass index before pregnancy was categorized into four groups according to the criteria by the World Health Organization: underweight (<18.50 kg/m^2^), normal weight (18.50–24.99 kg/m^2^), pre-obese (25.00–29.99 kg/m^2^), obese (≥30.00 kg/m^2^). Parity was defined as the number of previous live births and stillbirths while maternal smoking was defined as any smoking around the time of the last menstrual period. Chronic diseases included diabetes, epilepsy, asthma, and hypertension.

### Statistical analysis

Statistical analyzes were performed by using SAS (Statistical Analysis System) version 9.2 (SAS Institute, Inc., Cary, North Carolina) for Windows. Data were described as percentages or means with interquartile ranges (IQRs). The chi-square test was used to test for group difference in categorical variables. All P values were two-sided and values <0.05 were considered statistically significant. Associations of preconception folic acid use with the various maternal characteristics were estimated by prevalence ratios with 95 % confidence intervals (CIs) using log-binomial regression models with the *log-link* function. All prevalence ratios were estimated by crude models, as well as after adjustment for maternal age, parity, maternal education, marital status, national citizenship (here: Italian), pregnancy intention, preconception health visit, infertility treatment, and maternal smoking before pregnancy. All covariates have previously been strongly related to maternal folic acid supplement use. Additionally, the correlation between binary outcomes within the same maternity clinic was taken into account by using generalized estimating equations methodology, assuming an exchangeable working correlation structure. Women with missing data on covariates were excluded in multiple regression analyzes.

## Results

Of the 2,189 participating women, the mean maternal age was 33 (IQR: 29–37; range 13–50) years; only 6 % were younger than 25 years while 40 % were older than 35 years (Table 1). We further estimated that 64 % of the women had intended their pregnancy and that 30 % had mistimed their pregnancy. Overall, 41 % of the women reported that they had requested a preconception health visit to a doctor/gynecologist whereas 7 % had reported that they had used an infertility treatment. Reported smoking prevalence before the onset of pregnancy was as high as 26 %.

**Table 1:** Characteristics of the study sample

Characteristic		No. of women^a^	
		*n*	*%*
Maternal age (years)			
	<25	130	5.9
	25–29	404	18.5
	30–34	724	33.1
	35–39	660	30.2
	40 or more	209	9.5
Parity			
	0	1,246	56.9
	1	730	33.3
	2	171	7.8
	3 or more	42	1.9
Educational level			
	Primary school	331	15.1
	Secondary school	960	43.9
	University	890	40.7
Marital status			
	No partner	213	9.7
	Married/cohabiting	1,957	89.4
Citizenship			
	Non-Italian	319	14.6
	Italian	1,867	85.3
Working activity			
	Studying	106	4.8
	Working	1,683	76.9
	Working at home	283	12.9
	Not working	94	4.3
Pregnancy intention			
	Unintended	121	5.5
	Mistimed	646	29.5
	Intended	1,392	63.6
Preconception health visit			
	No	1,266	57.8
	Yes	907	41.4
Infertility treatment			
	No	2,001	91.4
	Ovulation inductor use, no ART	31	1.4
	ART	128	5.8
Maternal smoking before pregnancy			
	No	1,614	73.7
	Yes	575	26.3
BMI before pregnancy			
	Underweight	180	8.2
	Normal weight	1,573	71.9
	Pre-obese	303	13.8
	Obese	122	5.6
Chronic disease			
	No	1,959	89.5
	Yes	208	9.5

*Abbreviation*: *ART* assisted reproductive technology, *BMI* body mass index

^a^Percentages do not add to 2,189 and 100 % due to missing data: 62 women on maternal age, 8 women on educational level, 19 women on marital status, 3 women on citizenship, 23 women on working activity, 30 women on pregnancy intention, 16 women on preconception health visit, 29 women on infertility treatment, 11 women on body mass index before pregnancy, and 22 women with chronic disease

**Fig. 1:**
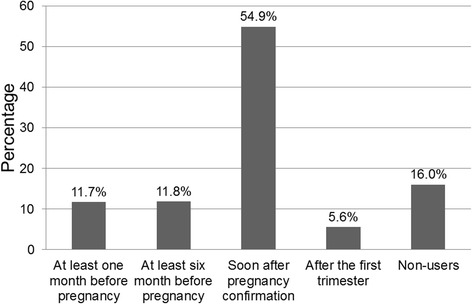
The prevalence of folic acid supplement users according to time periods of use (*n* = 2,189). Time periods of use were defined as at least one month before the last menstrual period to the end of the first trimester, at least six months before the last menstrual period to the end of the first trimester, soon after pregnancy confirmation to the end of the first trimester, after the first trimester of pregnancy, or no use at all

Overall, 84 % (*n* = 1,838) of the participating women had taken folic acid supplements at some time point before and/or during pregnancy while only 23.5 % (*n* = 515) of the participants had reported the use of folic acid supplements before the onset of pregnancy. Specifically, 11.7 % (*n* = 256) of all women had initiated use, at least, one month before pregnancy, 11.8 % (*n* = 259) had initiated use, at least, six months before pregnancy, 54.9 % (*n* = 1201) had initiated after pregnancy confirmation during the first trimester, and 5.6 % (*n* = 122) reported starting folic acid use after the first trimester of pregnancy (Fig. 1).

For all subgroups of women, intake of folic acid use was low during the preconception period and highest after pregnancy confirmation (Table 2). In particular, the initiation of supplement use in women younger than 25 years of age was only 5.4 % before pregnancy and 63 % soon after pregnancy confirmation.

**Table 2:** Prevalence of folic acid supplement users according to maternal characteristics and time periods of use^a^

Characteristic		No. of women	Folic acid supplement users, %^b^			
			At least one month before pregnancy (*n* = 256)	At least six months before pregnancy (*n* = 259)	Soon after pregnancy confirmation (*n* = 1,201)	After the first trimester (*n* = 122)
Maternal age (years)						
	<25	130	5.4	0	63.1	7.7
	25–29	404	11.4	7.4	58.7	4.7
	30–34	724	13.5	10.9	54.4	6.2
	35–39	660	12.3	15.8	51.1	5.2
	40 or more	209	10.5	19.1	53.1	4.8
Parity						
	0	1,246	12.4	14.4	52.8	4.5
	1	730	11.6	8.6	58.8	6.4
	2	171	8.2	8.8	55.0	9.4
	3 or more	42	4.8	2.4	47.6	7.1
Educational level						
	Primary school	331	3.9	5.4	53.5	8.2
	Secondary school	960	11.3	10.2	56.3	6.9
	University	890	15.1	16.1	54.2	3.3
Marital status						
	No partner	213	5.6	6.6	62.9	7.5
	Married/cohabiting	1,957	12.3	12.5	54.2	5.4
Citizenship						
	Non-Italian	319	8.2	6.0	53.0	10.0
	Italian	1,867	12.3	12.9	55.2	4.8
Working activity						
	Studying	106	10.4	7.5	61.3	5.7
	Working	1,683	12.4	12.7	55.0	5.4
	Working at home	283	8.5	8.8	53.7	6.0
	Not working	94	10.6	13.8	50.0	7.4
Pregnancy intention						
	Unintended	121	4.1	2.5	57.0	9.1
	Mistimed	646	4.3	4.0	62.4	7.3
	Intended	1,392	15.8	16.4	51.1	4.4
Preconception health visit						
	No	1,266	5.1	5.0	63.6	6.8
	Yes	907	21.2	21.6	42.9	3.9
Infertility treatment						
	No	2,001	11.0	9.9	57.0	5.5
	Ovulation inductor use, no ART	31	6.5	41.9	32.3	12.9
	ART	128	22.7	35.2	30.5	2.3
Maternal smoking before pregnancy						
	No	1,614	12.5	13.1	53.5	5.4
	Yes	575	9.4	8.3	58.6	6.1
BMI before pregnancy						
	Underweight	180	11.1	12.2	53.9	6.1
	Normal weight	1,573	12.4	12.3	54.2	5.6
	Pre-obese	303	11.2	9.9	59.4	4.3
	Obese	122	5.7	9.8	54.9	6.6
Chronic disease						
	No	1,959	11.2	11.0	55.4	5.8
	Yes	208	14.4	19.7	50.0	3.8

*Abbreviation*: *ART* assisted reproductive technology, *BMI* body mass index

^a^Time periods were defined as (a) at least one month before the last menstrual period to the end of the first trimester, (b) at least six months before the last menstrual period to the end of the first trimester, (c) soon after pregnancy confirmation to the end of the first trimester, (d) after the first trimester of pregnancy, or (e) no use at all

^b^Percentage of “no use at all” not shown in the table

Preconception folic acid use was reported by 23.5 % (*n* = 515) of the participants. Of these, 479 (93 %) women had taken folic acid supplements on a daily basis as recommended by the health authorities, 25 (4.9 %) women had taken the supplements 4–6 times a week while 6 (1.2 %) women had taken them 1–3 times a week. Five women (0.9%) did not report the frequency of use.

Unadjusted and adjusted regression analyses showed that age over 25 years, being pregnant for the first time, having a university degree/secondary school, being married/cohabiting, intending the pregnancy, requesting preconception health visit to a doctor/gynecologist, using infertility treatments, and having chronic diseases were all statistically significant determinants of preconception folic acid use (Table 3). Italian citizenship, ovulation inductor use, and non-smoking before pregnancy were not significant determinants after adjustment for covariates. Working status and body mass index before pregnancy were insignificant determinants both before and after covariate adjustments (Table 3).

**Table 3:** Prevalence and association of preconception folic acid supplement according to reported maternal characteristics

Characteristic		No. of women	No. of supplement users^a^		Crude^b^		Adjusted^c^	
					Prevalence ratio	95 % confidence interval	Prevalence ratio	95 % confidence interval
		*N*	*n*	*%*				
Maternal age (years)								
	<25^d^	130	7	5.4	0.23	0.07, 0.80	0.31	0.12, 0.80
	25–29	404	76	18.8	0.78	0.60, 1.01	0.94	0.79, 1.11
	30–34	724	177	24.4	1		1	
	35–39	660	185	28.0	1.13	1.03, 1.23	1.05	0.96, 1.15
	40 or more	209	62	29.7	1.18	1.03, 1.36	0.98	0.85, 1.14
Parity								
	0^d^	1,246	335	26.9	1		1	
	1	730	148	20.3	0.74	0.63, 0.86	0.86	0.77, 0.95
	2	171	29	17.0	0.65	0.56, 0.76	0.82	0.57, 1.18
	3 or more	42	3	7.1	0.24	0.08, 0.72	0.66	0.29, 1.51
Educational level								
	Primary school^d^	331	31	9.4	1		1	
	Secondary school	960	206	21.5	1.99	1.73, 2.29	1.69	1.46, 1.95
	University	890	277	31.1	2.80	2.41, 3.26	1.90	1.62, 2.22
Marital status								
	No partner^d^	213	26	12.2	1		1	
	Married/cohabiting	1,957	485	24.8	2.26	1.86, 2.75	1.44	1.21, 1.72
Citizenship								
	Non-Italian^d^	319	45	14.1	1			
	Italian	1,867	470	25.2	2.32	1.21, 4.42	1.40	0.99, 1.97
Working activity								
	Studying^d^	106	19	17.9	1		1	
	Working	1,683	422	25.1	1.47	0.89, 2.42	1.00	0.73, 1.36
	Working at home	283	49	17.3	1.15	0.68, 1.96	1.10	0.77, 1.58
	Not working	94	23	24.5	1.49	0.73, 3.06	1.31	0.88, 1.97
Pregnancy intention								
	Unintended^d^	121	8	6.6	1		1	
	Mistimed	646	54	8.4	1.34	0.79, 2.28	1.02	0.61, 1.70
	Intended	1,392	448	32.2	4.87	2.51, 9.43	2.40	1.14, 5.05
Preconception health visit								
	No^d^	1,266	127	10.0	1		1	
	Yes	907	388	42.8	4.21	3.09, 5.72	2.90	2.21, 3.82
Infertility treatment								
	No^d^	2,001	418	20.9	1		1	
	Ovulation inductor use, no ART	31	15	48.4	2.11	1.35, 3.29	1.00	0.62, 1.60
	ART	128	74	57.8	2.75	2.08, 3.64	1.35	1.08, 1.68
Maternal smoking before pregnancy								
	No^d^	1,614	413	25.6	1		1	
	Yes	575	102	17.7	0.70	0.59, 0.82	0.92	0.79, 1.07
BMI before pregnancy								
	Underweight^d^	180	42	23.3	1		1	
	Normal weight	1,573	389	24.7	1.11	0.89, 1.38	0.97	0.75, 1.25
	Pre-obese	303	64	21.1	1.01	0.81, 1.27	0.97	0.75, 1.26
	Obese	122	19	15.6	0.77	0.53, 1.11	0.84	0.54, 1.31
Chronic disease								
	No^d^	1,959	436	22.3	1		1	
	Yes	208	71	34.1	1.47	1.17, 1.85	1.22	1.09, 1.37

*Abbreviation*: *ART* assisted reproductive technology, *BMI* body mass index

^a^Any supplement use that started before the last menstrual period

^b^Crude prevalence ratio was estimated by log-binomial regression models

^c^Adjusted for maternal age, parity, maternal education, marital status, Italian citizenship, pregnancy intention, preconception health visit, infertility treatment, and maternal smoking before pregnancy

^d^Reference category

**Fig. 2:**
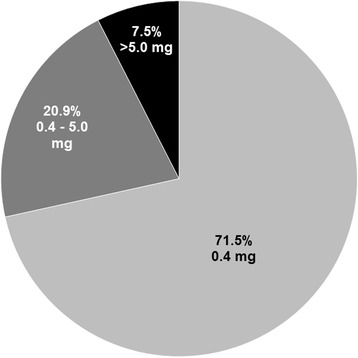
The distribution of reported folic acid dose (mg) among preconception supplement users (*n* = 515). Preconception folic acid supplement use was defined as any use of folic acid supplements that had started before the last menstrual period

Of the 515 women who had initiated use of folic acid-containing supplements before the onset of pregnancy, we had exact folic acid dose information on 506 women (98.2 %). Of these, 71.5 % had taken the usual dose of 0.4 mg, 20.9 % had taken 0.4 mg to 5.0 mg while 7.5 % had taken more than 5.0 mg (Fig. 2).

One-third of the women (*n* = 712) reported that they had both intended their pregnancy and had requested a preconception health visit to a doctor/gynecologist (Table 4). These women were eight times more likely to start folic acid supplementation before pregnancy compared with those who did not intend and did not request a preconception health visit (48.6 versus 4.8 %; adjusted prevalence ratio = 7.90 [95 % CI: 4.62, 13.5]).

**Table 4:** Prevalence and association of preconception folic acid supplement use according to status of preconception health visit to a doctor/gynecologist and status of pregnancy intention

Preconception health visit	Pregnancy intention^a^	No. of women^b^	No. of supplement users^c^		Crude^d^		Adjusted^e^	
					Prevalence ratio	95 % confidence interval	Prevalence ratio	95 % confidence interval
		*n*	*n*	*%*				
No^f^	No^f^	584	28	4.8	1.00		1.00	
No	Yes	659	97	14.7	2.98	1.93, 4.61	2.87	1.72, 4.79
Yes	No	188	39	20.7	4.35	2.33, 8.12	4.08	2.29, 7.28
Yes	Yes	712	346	48.6	9.69	5.68, 16.5	7.90	4.62, 13.5

^a^Status of pregnancy intention was defined as intended (yes) and unintended/mistimed (no)

^b^Data were missing for 30 women on pregnancy intention and 16 women on preconception health visit

^c^Any supplement use that started before the last menstrual period

^d^Crude prevalence ratio was estimated by log-binomial regression models

^e^Adjusted for maternal age, parity, maternal education, marital status, Italian citizenship, infertility treatment, and maternal smoking before pregnancy

^f^Reference category

Of 188 pregnancies that were not planned but in which the mother had consulted a doctor/gynecologists on preconception health advice (Table 4), 92 % (173/188) were classified as mistimed. The prevalence of folic acid supplement use in these 173 mistimed pregnancies was 21.4 % (not shown).

Finally, the estimated prevalence of preconception folic acid supplement use in the specific group of resourceful women (those who aged over 25 years, had a university degree, were married/cohabiting, had Italian citizenship, intended their pregnancy, requested a preconception visit) was 58.0 % [95 % CI: 52 %, 64 %], which was twice as high as the preconception prevalence of 23.5 % estimated for the total population.

## Discussion

Our study showed that 84 % of women had taken folic acid supplements at some time point before and/or during the pregnancy but that only 23.5 % of the participants had initiated use before the onset of pregnancy. In general, the percentage of initiation of folic acid supplement use was low before the onset of pregnancy and highest soon after pregnancy confirmation. This pattern was seen in all subgroups of women and suggests that the majority of women acquire folic acid information firstly after the onset of pregnancy. The most important determinants of folic acid use before the onset of pregnancy were preconception health visit to a doctor and pregnancy intention.

This is among the largest studies to examine the prevalence and determinants of maternal preconception folic acid supplement use in Italy. Strengths of the study include the detailed data on frequency, dose, and timing of folic acid supplement use, the analysis of a large number of maternal characteristics as determinants of supplement use, and the multicenter design in which data were collected through seven maternity clinics from six regions in Italy (three in the north, three in the center, and one in the south). However, our study sample may not be representative of all women giving birth in Italy. Women who did not comprehend the Italian language, those who delivered before 37 weeks of gestation or had an infant with birth weight below 2,500 g were not invited to participate. In addition, mothers with the lowest educational level may have been underrepresented in our study [27]. As these factors have been related to low use, our study might have overestimated the prevalence of preconception folic acid supplement use. Our prevalence estimate is still close to that reported in a previous study, which was 25.4 % in Italians [23].

Our findings on determinants of preconception folic acid use are generally consistent with those found in numerous other studies. Higher maternal age, having higher education, being married/cohabiting, having lower parity, using infertility treatments, and having a chronic disease have all been identified as statistically significant determinants of preconception folic acid use in previous studies [15–19]. In contrast to some findings, we did not identify national citizenship [17] and maternal smoking before pregnancy [15, 16] as significant independent factors after adjusting for covariates. Also, the association of ovulation inductor use was substantially attenuated after covariate adjustments, particularly after adjustment of preconception health visit and pregnancy intention (not shown). Consistent with two other large studies [15, 16], prepregnancy body mass index was not important determinants of preconception folic acid use in our study, neither was working status.

We identified preconception health visit to a doctor/gynecologist and pregnancy intention as the most important determinants of preconception folic acid supplement use. Obviously, women who intend their pregnancy or seek preconception advice from their doctor before pregnancy may be more informed on the benefits and timing of folic acid. Still, only 48.6 % (346/712) of all those who had requested preconception health visit and also had intended their pregnancy were using folic acid before pregnancy (see Table 4). An explanation for the low proportion of preconception folic acid users in this highly motivated group may be that recommendations on folic acid supplement use were not sufficiently provided to women by their doctors or that, in spite of sufficient information, women forgot or choose not to follow the doctor’s advice. These and other specific reasons for not taking folic acid supplements despite pregnancy planning and preconception health visits should be further investigated in studies to gain new insight on how to improve supplement use in these women [14].

As many as 27 % (584/2,189) women did not intend their pregnancy and did not request a preconception health visit (see Table 4). Of these, only 5 % (28/584) used folic acid-containing supplements before the onset of pregnancy. Clearly, information on the benefits of folic acid had not reached out to this large group of women, questioning the efficiency of the official recommendations to fertile women. To improve folate status in fertile women and particularly in women with unintended pregnancies, many countries, including the United States and Canada, have decided to fortify their foods with folic acid. In these countries, a risk reduction of neural tube defects has been reported [4–7]. Many countries refrain mandatory folic acid fortification, partly because of the risk of masking anemia related to a B12 deficiency in the elderly and partly because of unknown cancer risks in the non-pregnant population [9]. Recent results, however, show that folic acid in fortified foods and supplements does not interfere with vitamin B12 metabolism at the cellular level in a healthy population [28]. Additionally, a recent meta-analysis showed no significant risk (reduced or increased) of overall or site-specific cancers in nearly 50,000 individuals from 13 different randomized clinical trials of folic acid supplement use [29]. Given that Italy does not fortify food with folic acid and as many as 36 % mothers do not plan their pregnancy [25], new effective strategies for increasing supplement use in these pregnancies are strongly needed.

Of 188 pregnancies that were not planned but in which the mother had consulted a doctor/gynecologists on preconception health advice (see Table 4), 92 % (173/188) were classified as mistimed. This suggests that many women seeking preconception health advice had not necessarily decided to become pregnant at the time of the visit, but were open to the possibility of becoming pregnant in the near future. The prevalence of folic acid supplement use in these women was only 21.4 % and might be substantially improved by more direct measures by their doctors.

Folates occur naturally in foods, predominantly in green leafy vegetables, fruits, and cereal products. Although the typical Mediterranean diet constitutes a large portion of vegetables and fruits rich in folate it is uncertain whether this contributes to improved blood folate levels in Italian fertile women. In the European Prospective Investigation into Cancer and Nutrition cohort, women in South Europe, including Italy, appeared to have higher plasma folate concentrations than those in the northern, but not in the more central regions of Europe [30]. Furthermore, in a recent Italian study excluding vitamin B supplement users [31], only a small portion of women in childbearing age reached adequate levels of serum folate and red blood cell folate according to estimated thresholds for neural tube defect prevention [31, 32]. In light of this, supplementation with folic acid appears to be an important aspect also in countries with Mediterranean diets.

## Conclusions

In conclusion, like many women in other countries, many Italian women start folic acid supplement use too late with respect to neural tube defect prevention. Particularly, women who do not plan their pregnancy and do not request a preconception health visit to the doctor before the pregnancy have among the lowest prevalence of preconception folic acid use. Increased folic acid promotion during preconception health visits combined with an even higher rate of both planned pregnancies and preconception health visits might help improve supplement use in Italian women. Alternatively, mandatory food fortification with folic acid might be an option for improving folate status, but more information on the overall intake of folate from foods and supplements in Italy is needed before considering such interventions.

## Abbreviations

ART, assisted reproductive technologyl; BMI, body mass index; CI, confidence interval; IQR, interquartile range
